# A "Close" Time for Emotional Givers

**Published:** 1914-11-14

**Authors:** 


### A "CLOSE" TIME FOR EMOTIONAL GIVERS.

Is it not time for those responsible for the innumerable subscription funds which the war has called forth to cry a halt? The method of securing such a result is simple, as experience teaches. Within the last few days we have had several inquiries as to whether the Prince of Wales's Relief Fund is now closed, and what and where those who have collected funds and have them in hand are to do or to send them. The explanation is simple. For some weeks the daily papers contained a column or more every morning concerning the Prince of Wales's Relief Fund. To-day Press notices of the kind have almost disappeared. So far as the Prince of Wales's Fund is concerned, £4,000,000 would seem to us to be an ample contribution from the public for the present. It would be a calamity if the resources of the Fund were to be devoted to the relief of what is called distress during the winter months in this country. At the present time there is no distress in any proper meaning of the term, and yet hundreds of thousands of pounds are stated to have been spent in relief already. We hope it is not true, as stated, that the direction of the relief side of the Prince of Wales's Fund is at present controlled by the President of the Local Government Board and his department. If this is even largely true, we fear that that Fund so administered will result in doing an immensity of harm.

The present war has united all classes, and this unity of purpose and desire to be neighbourly should be utilised by statesmanship to destroy Bumble for ever. The procedure first proposed by the Prince of Wales's Fund and the notice issued by the Local Government Board in regard to the allowances to be paid to the wives and dependants of our sailors and soldiers revealed a depth of administrative incapacity which shocked the whole nation. Happily the action taken secured the independence of the wives and children of our sailors and soldiers, and the Prince of Wales's Relief Fund must now be freed absolutely from Local Government Board control, or it were better that it had never been brought into existence. The resources of the Prince of Wales's Relief Fund should be husbanded, so that a large proportion of them may be kept available for the years of the war and after. If some two and a half millions are to be handed over to the Local Government Board for the relief of so-called distress, the whole of it will probably be exhausted and wasted in a few weeks. The way in which the wives and dependants of our soldiers were treated and handled by people connected with this Fund in its early days constitutes one of the saddest examples of how not to do it which even Bumble has produced.

But apart from the Prince of Wales's Fund, the time has come when it is necessary to point out that givers must be prepared to continue to contribute year by year during the three years at least which it is expected the war will extend to, and must also husband their resources, because it is probable that the distress, in the right meaning of that word, will not really commence until the war terminates. There are proofs that the need for philanthropic effort and neighbourly action has been greatest invariably after a great war. We hope, however, the grants which are to be made by the Government to the permanently disabled and to the dependants of those who are slain may diminish the after-distress resulting from the present war. Still, we feel it to be our duty to cry a halt so far as the more emotional gifts and funds are concerned. For the time has come to put much energy into the provision in fuller measure for the hospitals and kindred institutions, the work of which must be upheld and adequately supported or grave injury to the people of these islands may result. At present the Voluntary Hospitals are providing more and more accommodation for the wounded, although there is no sufficient evidence that the money required is being sent to the hospitals. This is a matter the public should attend to at once.

